# c-Jun Proto-Oncoprotein Plays a Protective Role in Lung Epithelial Cells Exposed to Staphylococcal α-Toxin

**DOI:** 10.3389/fcimb.2018.00170

**Published:** 2018-05-25

**Authors:** Alejandro J. Moyano, Ana C. Racca, Gastón Soria, Héctor A. Saka, Verónica Andreoli, Andrea M. Smania, Claudia Sola, José L. Bocco

**Affiliations:** ^1^Departamento de Bioquímica Clínica, Facultad de Ciencias Químicas, Universidad Nacional de Córdoba, Córdoba, Argentina; ^2^Centro de Investigaciones en Bioquímica Clínica e Inmunología (CIBICI), CONICET, Universidad Nacional de Córdoba, Córdoba, Argentina; ^3^Departamento de Química Biológica Ranwel Caputto, Facultad de Ciencias Químicas, Universidad Nacional de Córdoba, Córdoba, Argentina; ^4^Centro de Investigaciones en Química Biológica de Córdoba, CONICET, Universidad Nacional de Córdoba, Córdoba, Argentina

**Keywords:** c-Jun, *Staphylococcus aureus*, α-toxin, α-hemolysin, bacterial pore-forming toxins

## Abstract

c-Jun is a member of the early mammalian transcriptional regulators belonging to the AP-1 family, which participates in a wide range of cellular processes such as proliferation, apoptosis, tumorigenesis, and differentiation. Despite its established role in cell survival upon stress, its participation in the stress response induced by bacterial infections has been poorly investigated. To study the potential role of c-Jun in this context we choose the widely studied α-toxin produced by *Staphylococcus aureus*, a pore-forming toxin that is a critical virulence factor in the pathogenesis of these bacteria. We analyzed the effect of α-toxin treatment in the activation, expression, and protein levels of c-Jun in A549 lung epithelial cells. Furthermore, we explored the role of c-Jun in the cellular fate after exposure to α-toxin. Our results show that staphylococcal α-toxin *per se* is able to activate c-Jun by inducing phosphorylation of its Serine 73 residue. Silencing of the JNK (c-Jun N-terminal Kinase) signaling pathway abrogated most of this activation. On the contrary, silencing of the ERK (Extracellular Signal-Regulated Kinase) pathway exacerbated this response. Intriguingly, while the exposure to α-toxin induced a marked increase in the levels of c-Jun transcripts, c-Jun protein levels noticeably decreased in the same time-frame as a consequence of active proteolytic degradation through the proteasome-dependent pathway. In addition, we established that c-Jun promoted cell survival when cells were challenged with α-toxin. Similarly, c-Jun phosphorylation was also induced in cells upon intoxication with the cytolysin produced by *Vibrio cholerae* in a JNK-dependent manner, suggesting that c-Jun-JNK axis would be a conserved responsive cellular pathway to pore-forming toxins. This study contributes to understanding the role of the multifaceted c-Jun proto-oncoprotein in cell response to bacterial pore-forming toxins, positioning it as a relevant component of the complex early machinery mounted to deal with staphylococcal infections.

## Introduction

*Staphylococcus aureus* is a primary health concern worldwide, being the etiological agent of diverse infections that span from mild to life-threatening (Chambers and Deleo, 2009; DeLeo et al., 2010). To achieve invasion and colonization of the host, *S. aureus* is able to display an assortment of virulence factors among which the *hla*-encoded α-toxin (also referred to as α-hemolysin) outstands as a focus of intense research because of its key role in the establishment of severe infections such as sepsis or pneumonia (Bubeck Wardenburg et al., 2007; Berube and Bubeck Wardenburg, 2013).

Monomers of Hla assemble to form a homoheptameric ring which pokes into the plasma membrane to function as a pore-forming toxin (PFT) (Gouaux, 1998; Menestrina et al., 2001; Montoya and Gouaux, 2003), an intercommunicating 2 nm channel that leads to the loss of cellular potassium and calcium homeostasis (Below et al., 2009; Eichstaedt et al., 2009; Kloft et al., 2009; Eiffler et al., 2016). In this sense, it has been observed that staphylococcal α-toxin *per se* is able to trigger cellular defense responses such as production of IL-6 and IL-8, autophagy, and activation of p38 and ERK signaling pathways, among many others (Ratner et al., 2006; Gonzalez et al., 2008; Below et al., 2009; Kloft et al., 2009; Mestre et al., 2010). Importantly, immediate downstream activation targets of MAPKs are the members of the AP-1 transcription complex—early responsive genes which have a primary role in central cellular functions such as proliferation, apoptosis, tumorigenesis, differentiation, and survival upon stress (Hess et al., 2004; Vesely et al., 2009; Shaulian, 2010; Yogev et al., 2010; Papoudou-Bai et al., 2016). One of the main components of AP-1 is c-Jun, the expression of which can be stimulated by a wide array of extracellular stimuli (Hess et al., 2004; Anzi et al., 2008; Vesely et al., 2009; Shaulian, 2010; Yogev et al., 2010; Papoudou-Bai et al., 2016). Moreover, these same stimuli can turn c-Jun transcriptionally active by inducing phosphorylation of its residues Ser63 and Ser73 in its N-terminal domain. This activation is generally mediated by one or more MAPKs, notably by JNK, ERK, and p38 signaling pathways (Dérijard et al., 1994; Morton et al., 2003; Humar et al., 2007; Meng and Xia, 2011). Interestingly, the three MAPKs are known to become phosphorylated when cells are exposed to α-toxin or other bacterial PFTs (Ratner et al., 2006; Aguilar et al., 2009; Below et al., 2009; Kloft et al., 2009; Porta et al., 2010; Gonzalez et al., 2011; Kao et al., 2011; Räth et al., 2013). However, apart from a single recent study (Kao et al., 2011), c-Jun has never been implicated as being part of the cellular response to PFTs such as the staphylococcal α-toxin.

By using A549 cells, derived from lung epithelium which is a target tissue of severe staphylococcal infections such as necrotizing pneumonia, we show that α-toxin induced early activation of c-Jun whilst prompting its proteasomal degradation. Furthermore, we demonstrate that c-Jun contributed to withstand the damage induced by α-toxin, thereby promoting cell survival. Thus, we expand the repertory of manifold functions known for c-Jun by enlisting it as part of the early cellular response to staphylococcal infections.

## Materials and methods

### Cell culture and treatment with staphylococcal α-toxin

Lung-derived epithelial A549 cells were routinely grown in Dulbecco's modified Eagle medium (DMEM; Gibco, Carlsbad, CA, USA) supplemented with 10% fetal bovine serum (FBS) (PAA Laboratories, Pasching, Austria) and antibiotics (100 U ml^−1^ penicillin and 100 μg ml^−1^ streptomycin; Gibco). Cells were then left overnight with serum-free medium and further treated or not with staphylococcal α-toxin (Sigma-Aldrich) 5 μg ml^−1^ in fresh medium with 10% FBS for the corresponding time period.

All laboratory procedures were performed according to the Laboratory Safety Standards. Staphylococcal α-toxin was handled following the provider's recommendations.

### siRNA-mediated knockdown

c-Jun and MAPKs were silenced in A549 cells using 100 nM of siRNA for c-Jun and JNK and, 25 nM for p38 and ERK (SignalSilence®, Cell Signaling Technology) that were transfected with Lipofectamine™ RNAiMAX (Invitrogen) following the manufacturer's recommendations. Controls were carried out using SignalSilence® Control siRNA (SCR). Subsequent to this, cells were exposed or not to the staphylococcal α-toxin followed by Western blot analyses.

### Western blotting

Western blots were carried out as described previously (Racca et al., 2011). Briefly, whole protein extracts of A549 cells were prepared in 5X Laemmli buffer containing 60 mM Tris-HCl pH 6.8, 10% glycerol, 2% sodium dodecyl sulfate (SDS), 1% 2-β-mercaptoethanol and 0.002% bromophenol blue. Total protein samples were separated on a 10% SDS-PAGE, and proteins were transferred to a nitrocellulose Hybond-ECL (Amersham Bioscience). The membranes were blocked in 5% non-fat milk in TBS (20 mM Tris-HCl, 150 mM NaCl pH 7.8), supplemented with 0.1% Tween-20 (TBS-T) 1 h at room temperature. Blots were incubated overnight with primary antibodies diluted in TBS-T at 4°C. The following antibodies were used: rabbit polyclonal anti-c-Jun (H-79; Santa Cruz Biotech.; 1:1000), rabbit monoclonal anti-phospho-c-Jun (Ser73) (#9164; Cell Signaling Technology; 1:1000), mouse monoclonal anti-β-actin (Sigma–Aldrich; 1:3000). After washing, the blots were incubated with horseradish peroxidase-conjugated donkey anti-rabbit or sheep anti-mouse IgG secondary antibodies (Amersham Bioscience; 1:5000) in TBS-T, at room temperature for 1 h. Protein-antibody complexes were visualized using an enhanced chemiluminescence detection system (SuperSignalWest Pico; Pierce) and exposed to Amersham Hyperfilm ECL. Ponceau staining (0.2% Ponceau, 3% tricloroactic acid, 3% sulfosalicilic acid) was used to verify protein transference from gel to nitrocellulose membrane. Band intensities were determined using Gel-Pro Analyzer software.

### Quantitative real time PCR

Total RNA was extracted from cultured cells at the indicated times using GeneJET RNA Purification Kit (Thermo Fisher Scientific). One microgram of total RNA was reverse-transcribed in a total volume of 20 μl using random primers (Invitrogen) and 50 U M-MLV reverse transcriptase (Promega Corp.). c-Jun specific primers RT-cJun-For (5′-GACGATGCCCTCAACGCC-3′) and RT-cJun-Rev (5′-AGGATCTTGGGGTTACTGTAGCC-3′) were manually designed with the assistance of the Netprimer software (PREMIER Biosoft International, Palo Alto, CA). Primer sequences were compared against the human genomic and transcript data base with the BLAST program (Altschul et al., 1997) at the NCBI Web site. Specific transcripts were quantified by real time qRT-PCR (ABI 7500 Sequence Detection System, Applied Biosystems) using the Sequence Detection Software v1.4. Experiments were performed using 1x SYBR Green PCR Master Mix (Applied Biosystems). The cycling conditions included a hot start at 95°C for 10 min, followed by 40 cycles at 95°C for 15 s and 60°C for 1 min. Specificity was verified by melting curve analysis and agarose gel electrophoresis. Fold change in gene expression was calculated according to the 2–ΔΔCt method (Livak and Schmittgen, 2001). Each sample was analyzed in triplicate. No amplification was observed in PCR reactions containing water or RNA samples incubated without reverse transcriptase during cDNA synthesis as template.

### Determination of c-Jun protein degradation

A549 cells were treated with the proteasome pharmacological inhibitor MG132 (40 μM) (Sigma–Aldrich) for 30 min prior to treatment with α-toxin. Additionally, In order to clean off the effect of newly synthesized c-Jun, cells were also treated with cycloheximide (CHX) 1 μg/ml, which inhibits protein synthesis by blocking translational elongation. The vehicle DMSO was used as control.

### Determination of dehydrogenase activity

Cell viability was evaluated by measuring dehydrogenase activity with the CellTiter 96® A_queous_ Non-Radioactive Cell Proliferation Assay kit (Promega, Madison WI, USA) following the manufacturer's instructions. Briefly, A549 cells were plated in triplicate wells of a 96-well plate at a density of 3 × 10^3^ cells/well. The cells were incubated for 24 h at 37°C. Cell viability was measured after incubation for 2 h with a combined MTS/PMS solution by reading the plates at 490 nm with a microplate reader (Bio-Rad, Hercules, CA, USA). Viability was calculated from the absorbance ratio between values obtained at the end point with respect to those measured in cells before treatment. All assays were repeated 4 times.

### Assessment of cell death by annexin V/Propidium iodide double staining assay

A549 cell were cultured in complete growth medium, silenced for c-Jun using siRNA and exposed to α-toxin. After different time points post incubation, cells were harvested, washed and suspended in cold PBS. The proportion of cells undergoing cell death was examined by double staining using FITC labeled Annexin V and propidium iodide, as instructed by the manufacturer's protocol. At least 10,000 cells were analyzed and quadrant analysis was performed using FlowJo software version 7.6.2 (Tree Star, Inc., Ashland, OR). Viable cells are refractory to both staining agents. Cells in early stages of apoptosis stained as Annexin V^+^/Propidium iodide^−^, whereas cells in late apoptosis stained as Annexin V^+^/Propidium iodide^+^. Cells Annexin stained V^−^/Propidium iodide^+^ are considered necrotic.

### Statistical analysis

Statistical analyses were performed using GraphPad Prism version 5.03 software (GraphPad Sofware, San Diego, California, USA). The data were analyzed by one-way or two-way ANOVA when appropriate followed by Dunnet's or Bonferroni's *post hoc* tests, respectively. Kruskal-Wallis statistics was used for nonparametric adjustments followed by Dunn's multiple comparison test. In all cases, p-values less than or equal to 0.05 were considered statistically significant.

## Results

### α-toxin induces c-Jun activation

Members of the AP-1 transcription factors are naturally activated via phosphorylation by a wide array of cellular and extracellular stimuli (Hess et al., 2004; Anzi et al., 2008; Vesely et al., 2009; Shaulian, 2010; Yogev et al., 2010; Papoudou-Bai et al., 2016). We wondered whether c-Jun, an early responsive transcription factor—could be activated after exposure of lung-derived cells to staphylococcal α-toxin.

For this purpose, we exposed A549 cells to α-toxin for different periods of time and followed the activation dynamics of c-Jun by examining its phosphorylation at its Ser73 residue, which mediates c-Jun activation. Thus, we analyzed cell extracts by Western blot using specific antibodies against this phosphorylated form of c-Jun.

**Figure 1:**
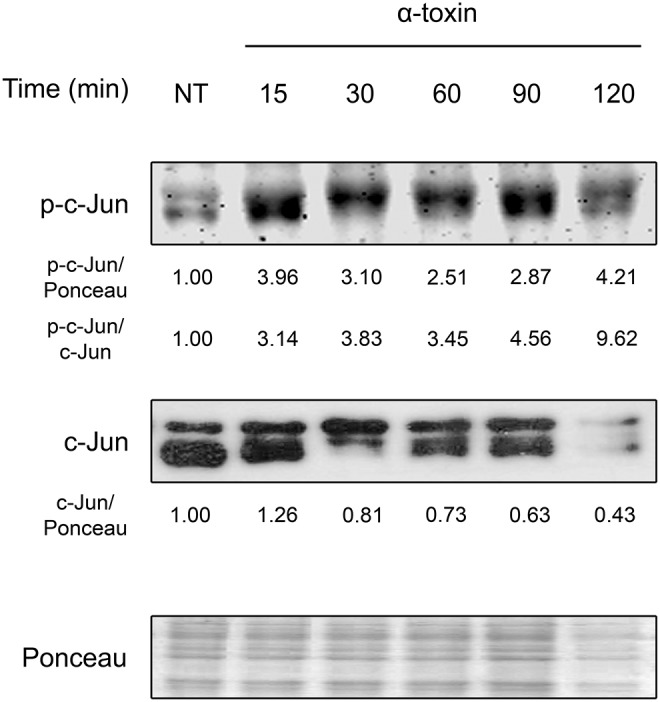
Activation of c-Jun by staphylococcal α-toxin. Proteins were extracted from cultures of lung epithelial A549 cells which had been treated with α-toxin (5 μg ml-1) for 15–120 min or left untreated (NT). Western Blots were performed with specific antibodies against the phosphorylated and total fractions of c-Jun. Ponceau staining of total transferred proteins were used as loading controls. The panels illustrate typical blots obtained. Below the panels, relative protein levels are shown, with NT values normalized to 1.

As shown in Figure 1 and Figure S1, staphylococcal α-toxin significantly induced c-Jun phosphorylation after only 15 min of exposure. Interestingly, this activation response was accompanied with a concomitant decrease in the levels of total c-Jun, which is further described later in this study. These results indicate that α-toxin *per se* is sufficient to produce early activation of c-Jun, suggesting that this transcription factor could play a role in the cellular response against staphylococcal infections. Furthermore, staphylococcal α-toxin is able to decrease the protein levels of c-Jun which might carry subsequent implications in cellular fate.

### The α-toxin induced phosphorylation of c-Jun occurs through the JNK pathway

**Figure 2:**
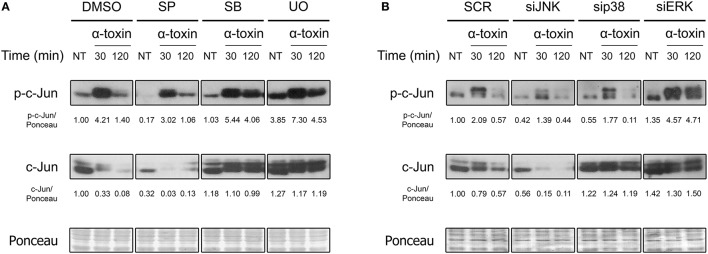
Role of MAPKs in the α-toxin-induced activation of c-Jun. **(A)** Lung epithelial A549 cells were pretreated with the JNK inhibitor SP600125 (SP) 30 μM, the p38 inhibitor SB203580 (SB) 10 μM, or with the MEK1/2-ERK inhibitor U0126 (U0) 10 μM for 2 h, with the carrier DMSO being used to obtain uninhibited controls; **(B)** MAPKs were silenced using specific siRNAs for JNK (100 nM), p38 (25 nM), and ERK (25 nM). Controls were carried out using SignalSilence® Control siRNA (SCR). Subsequent to inhibition/silencing, cells were exposed or not to the staphylococcal α-toxin (5 μg ml-1) for 30 and 120 min. Finally, the entire cell extracts were used for Western blotting by using specific antibodies against the phosphorylated and total fractions of c-Jun. Ponceau staining was carried out for loading controls. Pictures correspond to one representative assay of at least three independent experiments. NT, not treated. Below the panels, relative protein levels are shown, with NT values normalized to 1.

Members of the AP-1 transcription factors are naturally activated via phosphorylation by MAPKs. Since it has already been observed that bacterial pore forming toxins induce the phosphorylation of MAPKs (Ratner et al., 2006; Aguilar et al., 2009; Below et al., 2009; Kloft et al., 2009; Porta et al., 2010; Gonzalez et al., 2011; Kao et al., 2011; Räth et al., 2013), we wondered whether these could, in turn, mediate c-Jun phosphorylation after exposure to staphylococcal α-toxin. Thus, we evaluated the role of JNK, p38, and ERK MAPKs in this response process, being JNK the main activator of c-Jun upon several different stimuli (Meng and Xia, 2011). In this sense, we observed the activation response of c-Jun in A549 cells which had been previously treated with specific pharmacological inhibitors of JNK, p38, or ERK (Figure 2A, Figure S2A). Alternatively, we also evaluated their contribution in this process by silencing the three MAPKs with the use of siRNA (Figure 2B, Figures S2B, S3).

As shown in Figure 2 and Figure S2, pharmacological inhibition or knockdown of JNK by siRNA significantly reduced the phosphorylation of c-Jun in cells treated with α-toxin. On the contrary, basal and α-toxin-induced phosphorylation of c-Jun was increased in cells with knockdown or pharmacological inhibition of ERK. In fact, ERK inhibition/silencing also increased the levels of total c-Jun protein, counteracting the decrease observed at 120 min (Figure 2, Figure S2). A less clear effect was observed when the role of p38 was evaluated. While pharmacological inhibition of p38 produced a statistically non-significant increase in the activation as well as total protein levels of c-Jun, siRNA-derived knockdown of p38 produced no apparent modifications in the Ser73 phosphorylation. This could be probably attributed to off-target effects of the pharmacological p38 inhibitor. Alternatively, it is possible that even siRNA-mediated knock down levels of p38 that still conserve their intact catalytic capacities could be interfering in c-Jun phosphorylation.

Taken together, the results suggest that JNK is involved in the main signaling pathway that leads to α-toxin-induced activation of c-Jun. On the other hand, ERK seems to be carrying out a negative regulation on c-Jun in α-toxin-treated cells whereas the role of p38 is not clear.

### Staphylococcal α-toxin induces the degradation of c-Jun protein

As shown in Figure 1 and Figure S1, the levels of total c-Jun decreases drastically after 30–60 min of treatment with α-toxin. There are two main possible pathways to achieve this outcome. The first is a decrease in the amount of transcripts of c-Jun followed by the concomitant lesser synthesis of the c-Jun protein. The second is an alteration in the stability and further degradation of the c-Jun protein provoked by those α-toxin-derived stimuli.

**Figure 3:**
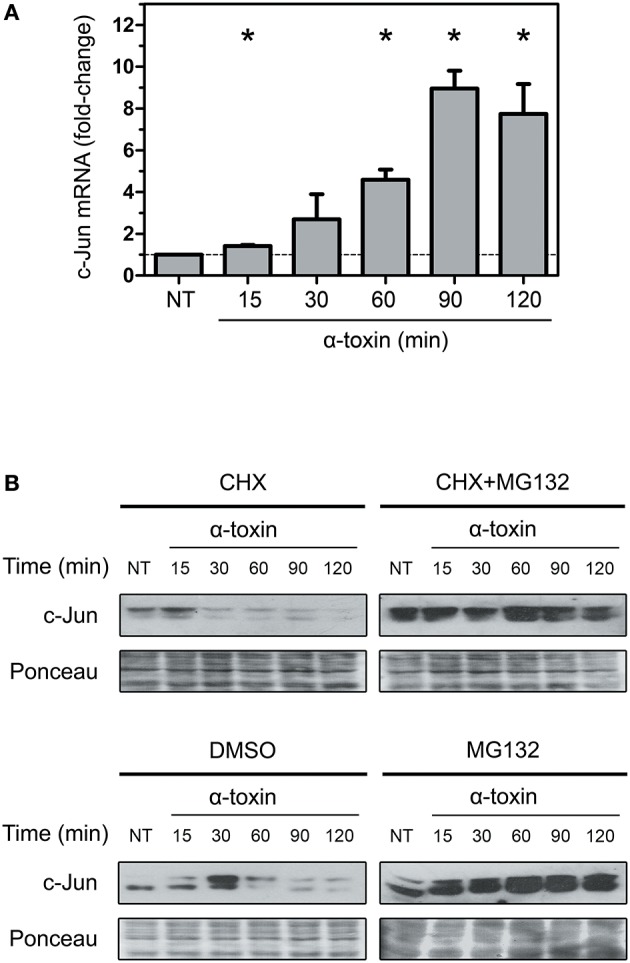
Effect of α-toxin on the expression and stability of c-Jun. **(A)** Total mRNA was extracted from A549 cells which had been treated with α-toxin (5 μg ml-1) for 15 to 120 min or left untreated (NT). Subsequently, the amounts of c-Jun transcripts were determined by Quantitative real time PCR. Measurements were carried out in triplicate for three independent experiments, and the results are expressed as means with their SEM. Statistically significant differences at *P* < 0.05 are identified by ^*^ (Kruskal-Wallis followed by Dunn's multiple comparison test). **(B)** A549 cells were treated with the translational inhibitor cycloheximide (CHX, 1 μg ml-1), the proteasome pharmacological inhibitor MG132 (40 μM) or, the combination of both for 30 min, with the carrier DMSO being used for control experiments. Subsequently, cells were exposed to α-toxin (5 μg ml-1) for 15 to 120 min or left untreated (NT). Western Blots were performed with specific antibodies against c-Jun. Ponceau staining of total transferred proteins were used as loading controls. The panels illustrate typical blots obtained from 4 independent experiments.

In order to test the first possibility, we performed real-time PCR to measure the amount of *c-jun* transcripts. We carried out these analyses by purifying RNA from A549 cells treated with α-toxin using the same time periods of exposure described above. Surprisingly, in clear opposition to the decrease observed in protein levels, the transcript levels of c-Jun increased steadily until 90 min of exposure to the toxin, followed by a slight decrease at 120 min (Figure 3A). This result cannot explain the observations on protein levels and puts forward the hypothesis of an increased protein instability and degradation.

To test the second alternative, we analyzed if α-toxin was able to induce degradation of c-Jun, thereby contributing to the reduction in the amount of protein observed. It has been established that elimination of c-Jun naturally occurs via polyubiquitination and subsequent degradation by the proteasome (Treier et al., 1994; Salvat et al., 1998). In this sense, we analyzed the effect of α-toxin on A549 cells which have been previously treated with cycloheximide (CHX) for protein synthesis inhibition, or CHX combined with MG132, a selective proteasome inhibitor. As shown in Figure 3B, the use of MG132 in addition to CHX rescued the levels of c-Jun in cells treated with α-toxin. Treatment of cells with MG132 alone rendered even more accumulation of c-Jun protein due to both, the contribution of *de novo* c-Jun biosynthesis and impaired degradation upon proteasome inhibition.

The decrease of c-Jun observed at longer exposures times (90–120 min) in CHX+MG132 treated cells could be due to partial inhibition of the proteasomal pathway by MG132 or, alternatively, due to other mechanisms of protein degradation.

These results suggest that a strong induction of protein degradation but not a reduced expression at mRNA level is the primary cause of the α-toxin-induced decrease observed in the protein levels of c-Jun.

### C-Jun plays a protective role upon α-toxin-mediated intoxication

**Figure 4:**
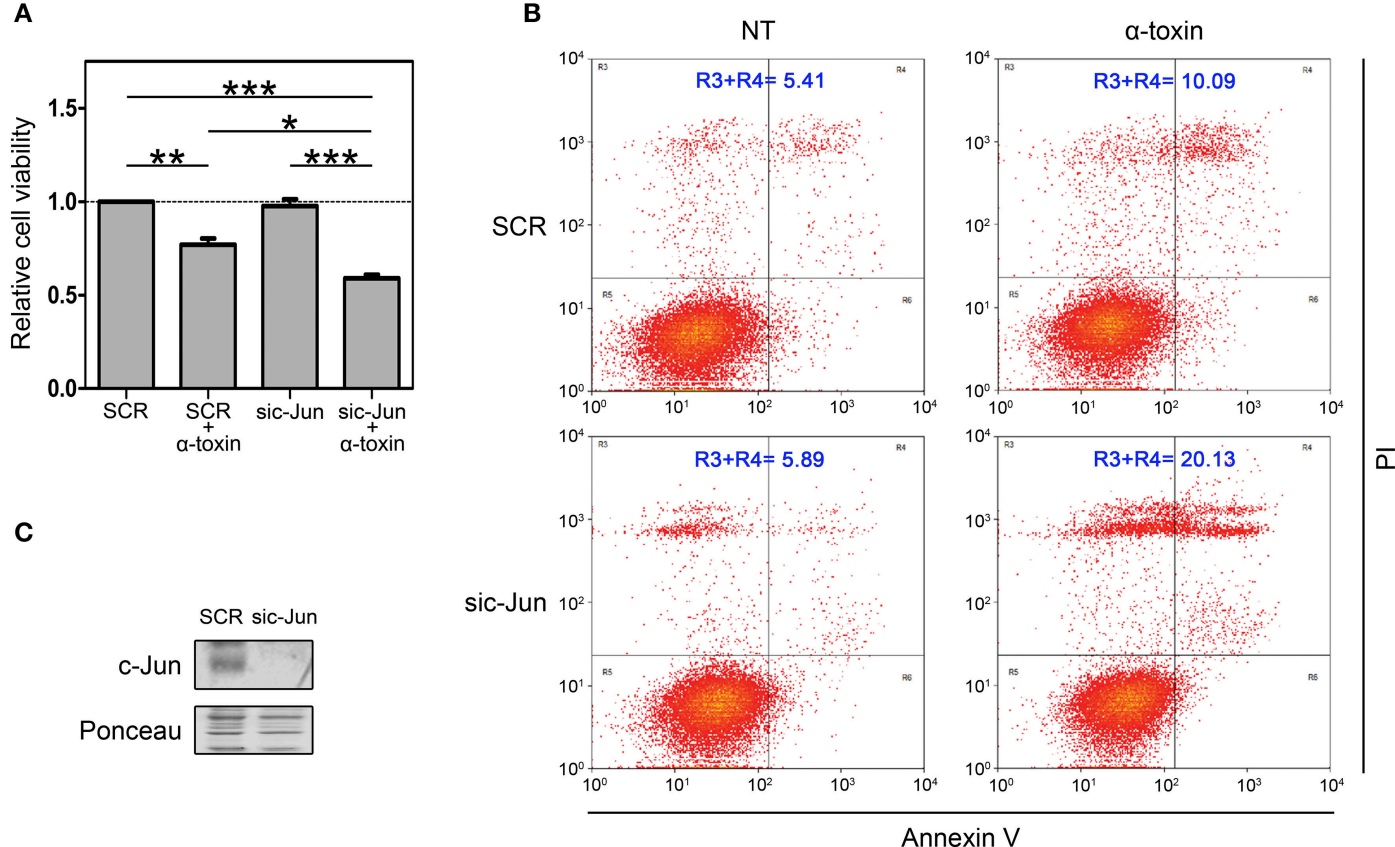
Role of c-Jun in the viability of cells treated with α-toxin. **(A)** Dehydrogenase activity assays: A549 cells with normal (SCR) or knockdown (sic-Jun) levels of c-Jun were exposed to α-toxin (5 μg ml-1) for 4 h or left untreated. Cell viability was measured after incubation for 2 h with a combined MTS/PMS solution by reading absorbance at 490 nm with a microplate reader (Bio-Rad, Hercules, CA, USA). Viability was calculated from the absorbance ratio between values obtained at the end point with respect to those measured in SCR untreated cells. Measurements were carried out in triplicate for 4 independent experiments, and the results are expressed as means with their SEM. Statistically significant differences at *P* < 0.001, *P* < 0.01, and *P* < 0.05 are identified by ^***^, ^**^, and ^*^, respectively (one-way ANOVA followed by Dunnet's *post hoc* test). **(B)** Flow-cytometry analyses: A549 cells with normal (SCR) or knockdown (sic-Jun) levels of c-Jun were exposed to α-toxin (5 μg ml-1) for 4 h or left untreated. Subsequently, cells were double stained with propidium iodide and the fluorescence-labeled annexin V followed by flow cytometric analyses to quantify cell death. One representative experiment of two is shown. **(C)** Knockdown of c-Jun was carried out using 25 nM of siRNA for c-Jun, using SignalSilence® Control siRNA (SCR) for controls. A representative Western Blot performed with specific antibodies against c-Jun is shown. Ponceau staining of total transferred proteins were used as loading controls.

In an initial attempt to investigate the biological contribution of c-Jun in the context of α-toxin exposure, we silenced c-Jun expression in A549 cells and we quantified dehydrogenase activity as a mitochondrial indicator of cell viability (Figure 4A). We compared the effect of α-toxin on cells with basal or knocked-down levels of c-Jun (Figure 4C). As shown in Figure 4A, cell viability upon treatment with α-toxin was significantly higher in those cells with normal levels of c-Jun compared to those in which c-Jun was silenced.

In order to reinforce this observation, we also quantified the induction apoptosis and necrosis by Annexin V/Propidium iodide double staining assay. In line with the results of dehydrogenase activity, when comparing cells with normal or silenced levels of c-Jun exposed to α-toxin we observed that knockdown of c-Jun increased the proportion of late apoptosis/necrosis (Figure 4B).

Taken together, the results from dehydrogenase activity and late apoptosis/necrosis quantification clearly indicate that c-Jun contributes to cope with the damage produced by the pore-forming α-toxin, thus promoting cell survival. Our results suggest that in the context of staphylococcal infections, c-Jun constitutes a part of the early-responsive repertory favoring cell survival.

## Discussion

In the present study we evaluated the effect of α-toxin, a main *S. aureus* virulence factor on c-Jun, a central early responsive cell cycle modulator that belongs to the AP-1 family of transcriptional regulators. c-Jun participates in a wide range of cellular functions such as proliferation, apoptosis, tumorigenesis and differentiation (Wisdom et al., 1999; Hess et al., 2004; Papoudou-Bai et al., 2016). In this work we expand this range by providing insights in a poorly explored field of c-Jun, which is its role in the context of bacterial infections. Our results indicate that the staphylococcal α-toxin *per se* is sufficient to produce early phosphorylation of c-Jun at its Ser73 residue, which has been profusely documented as a post-translational modification leading to an enhanced transcriptional activity of c-Jun. Noteworthy, this increased phosphorylation level is accompanied by a decrease in the amount of total c-Jun because of proteasomal degradation.

A549 cells derive from human lung adenocarcinoma, and as such they might show intrinsically unbalanced responses to different stimuli such as toxins. Interestingly, this c-Jun response seems not to be confined to a response of A549 cells to staphylococcal α-toxin, since own preliminary results showed that MEF cells underwent an equivalent c-Jun activation process along with a decrease in total protein levels of c-Jun after being exposed to *Vibrio cholera* cytolysin (VCC) (Figure S4), another PFT able to trigger severe pathogenic processes on eukaryotic cells (McCardell et al., 1985; Gutierrez et al., 2007; Saka et al., 2007, 2008; Khilwani and Chattopadhyay, 2015). Thus, this early phosphorylation-mediated activation of c-Jun could play an important role in the cell defense response upon intoxication with bacterial PFTs. In this sense, the results from dehydrogenase activity and Annexin V/Propidium iodide double staining clearly indicate that c-Jun contributes to cope with the damage produced by the pore-forming α-toxin favoring cell survival. In line with previous reported information, we observed that α-toxin mediated death, in cells with or knock-down levels of c-Jun involved mainly necrosis and late apoptosis/necrosis whereas apoptosis was almost undetected (Essmann et al., 2003). Coincidently, a previous study described a similar protective role for c-Jun when cells are exposed to two other bacterial pore forming toxins, namely CryB5 from *Bacillus thuringiensis* and streptolysin O from *Streptococcus pyogenes* (Kao et al., 2011). In addition, these authors also showed that both PFTs are able to increase the transcripts of c-Jun as we did observe here for α-toxin. Nevertheless, here we showed that the increase in transcripts induced by α-toxin was clearly opposite to the response at protein levels. It has been demonstrated that the *c-Jun* promoter and gene expression can be activated by its own c-Jun/AP1 protein product through an autocrine amplification loop mechanism (Angel et al., 1988b; Meng and Xia, 2011). In this sense, the α-toxin-induced activation of c-Jun protein could help to explain the increase of c-Jun mRNA levels. Concomitantly, c-Jun degradation could be part of a cell safeguard mechanism to avoid deleterious effects of prolonged c-Jun activation on cell physiology. Another exciting possibility is that the increased expression of c-Jun mRNA is the consequence of an early cell response aimed to cope with the damage triggered by α-toxin, which involves degradation of c-Jun protein as part of the pathogenic process.

Activation of c-Jun can be mediated by different MAPKs, depending on the stimulus (Dérijard et al., 1994; Morton et al., 2003; Humar et al., 2007; Meng and Xia, 2011). We observed that α-toxin-dependent activation of c-Jun occurs mainly via JNK. Accordingly, VCC-induced activation of c-Jun in MEF cells was also abrogated upon pharmacological inhibition of JNK (Figure S4). In agreement with the decrease in c-Jun observed after inhibition/silencing of JNK (Figure 2), it has been reported that phosphorylation of c-Jun on its Ser73 by JNK protects c-Jun from ubiquitination, thereby prolonging its half-life (Fuchs et al., 1996). Interestingly, JNK but not p38 was found to be a key regulator of the transcriptional and functional response to PFTs in a *C. elegans* model (Kao et al., 2011). On the other hand, ERK exerts a negative regulation on c-Jun. In relation to this, we have recently observed that silencing of ERK exacerbates the activation of JNK as part of a crosstalk phenomenon that occurs when cells are intoxicated with α-toxin (unpublished observation). This could help to explain the increase in the toxin-induced activation of c-Jun in ERK-depleted cells. However, another possibility is that ERK could be helping in the instability of c-Jun caused by α-toxin. In this sense, it has been observed *in vitro* that ERK is able to phosphorylate c-Jun at its Ser243 residue (Morton et al., 2003), which was in turn established to be required for GSK3-dependent phosphorylation of Thr239 and further polyubiquitination and degradation (Wei et al., 2005). Thus, it is tempting to speculate that the early activation followed by proteasomal degradation of c-Jun could serve to trigger the cell nuclear response while avoiding the positive effects of c-Jun on cell proliferation (Eferl and Wagner, 2003).

It has been reported that staphylococcal α-toxin is also able to modify the expression of c-Fos (Below et al., 2009; Kao et al., 2011), the main partner of c-Jun to compose the AP-1 transcription factor complex (Angel et al., 1988a; Harshman et al., 1988; Rauscher et al., 1988a,b). However, in opposition to the degradation of c-Jun that we observed here, α-toxin induces an increase in the protein levels of c-Fos (Below et al., 2009), with this induced expression being positively regulated by the MEK1/2-ERK pathway.

This study sheds light on a previously underexplored role of c-Jun as an early regulator of cellular response to bacterial PFTs. Undoubtedly, future transcriptome/proteome-based studies as well as deeper analyses of its post-translational modifications will be required to fully comprehend the role of c-Jun in the context of microbial infections.

## Author contributions

AM, AR, GS, VA, and HS performed experiments. AM, HS, AS, CS, and JB analyzed data. AM, HS, and JB designed the study. AM and JB wrote the paper.

### Conflict of interest statement

The authors declare that the research was conducted in the absence of any commercial or financial relationships that could be construed as a potential conflict of interest.
