# Supplementary material for: c-Jun Proto-Oncoprotein Plays a Protective Role in Lung Epithelial Cells Exposed to Staphylococcal α-Toxin
Source: Front Cell Infect Microbiol. 2018 May 25;8:170. doi: 10.3389/fcimb.2018.00170 (PMC5981160; doi:10.3389/fcimb.2018.00170)
Supplement: Supplementary file 1 [file Data_Sheet_1.pdf]

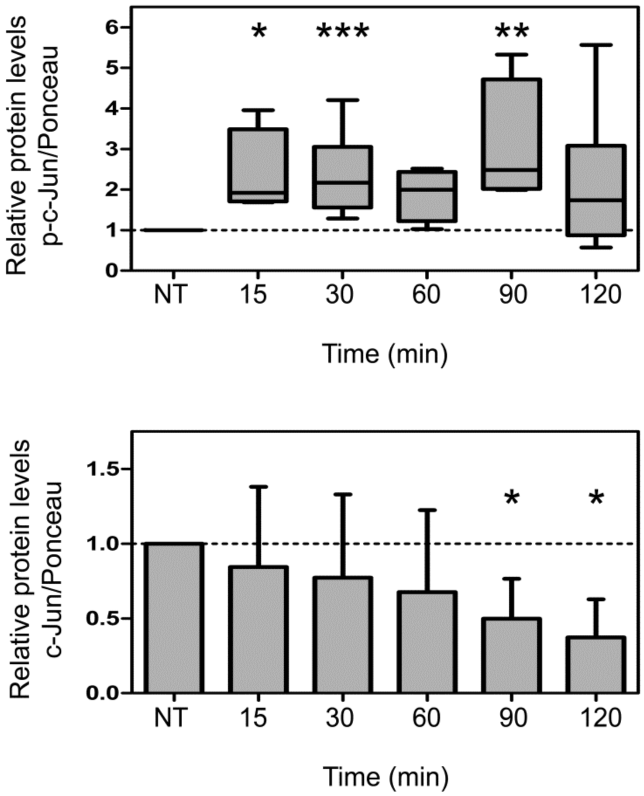

Fig. S1. Activation of c-Jun by staphylococcal α-toxin. Relative protein levels averaged over at least 3 independent experiments (means ± SD), with NT values normalized to 1. Statistically significant differences at P<0.001, P<0.01 and P<0.05 are identified by ***, ** and *, respectively (Kruskal-Wallis followed by Dunn's multiple comparison test and one-way ANOVA followed by Dunnet´s post hoc test for nonparametric or parametric adjustments, respectively).

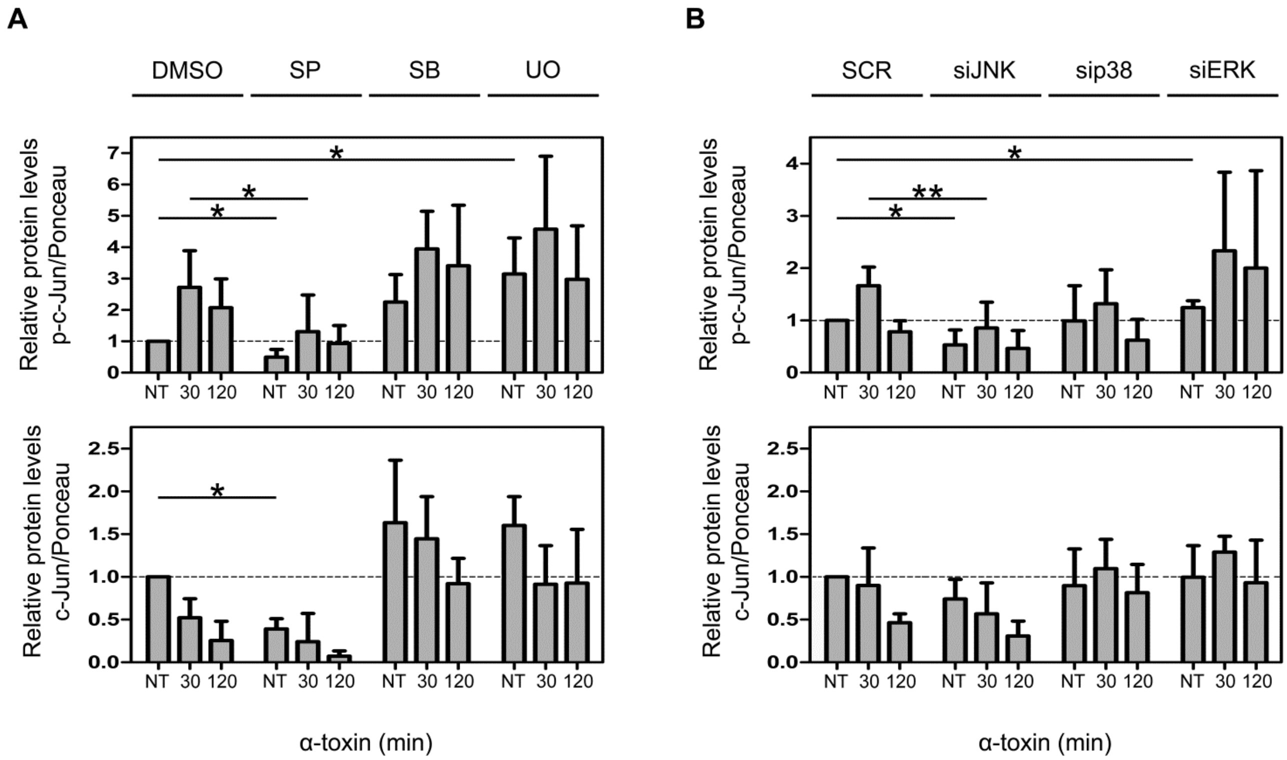

Fig. S2. Role of MAPKs in the α-toxin-induced activation of c-Jun. Plots show relative protein levels of at least three independent experiments described in Fig. 2 (means ± SD), with NT values normalized to 1. Statistically significant differences at $P<0.01$ and $P<0.05$ are identified by ** and *, respectively (two-way ANOVA followed by Bonferroni's post hoc test).

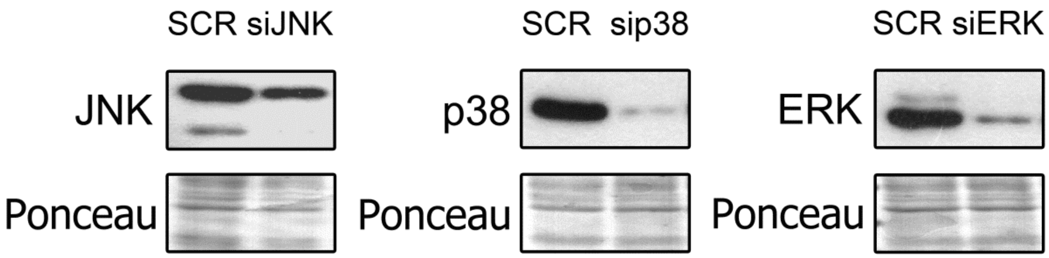

Fig. S3. Silencing of MAPKs. MAPKs were silenced in A549 cells using specific siRNAs for JNK (100nM), p38 (25nM) and ERK (25nM). Controls were carried out using SignalSilence® Control siRNA (SCR).Western Blots were perfromed using specific antibodies against JNK, p38 and ERK. Ponceau staining was carried out for loading controls.

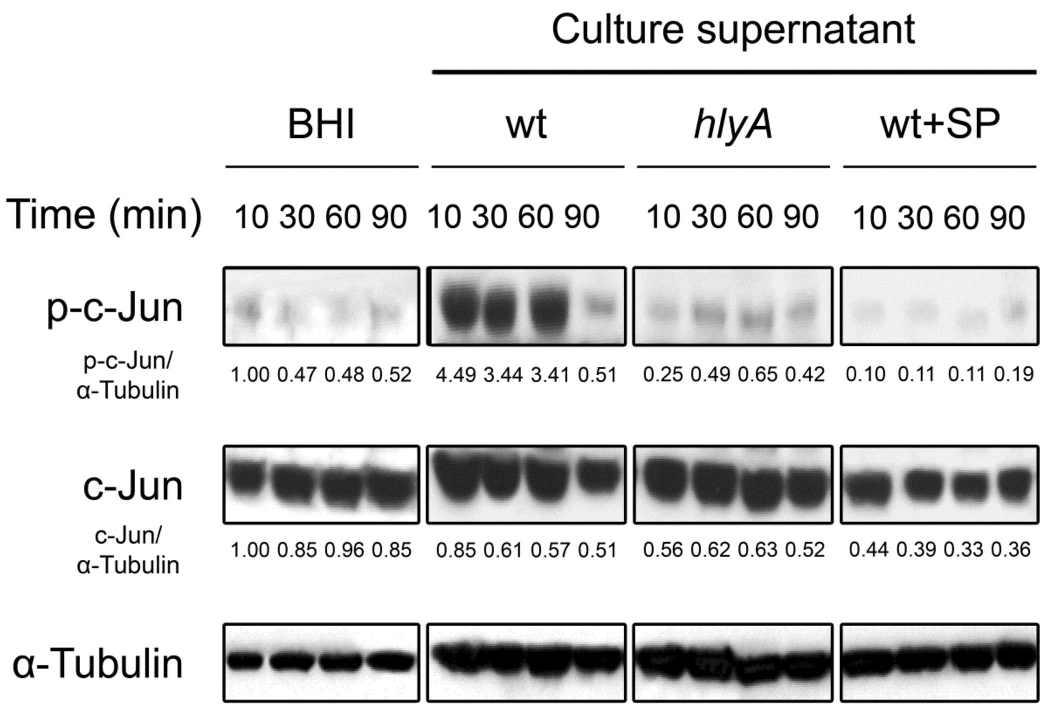

Fig. S4. Activation of c-Jun by *V. cholera* cytolysin (VCC). Proteins were extracted from cultures of MEF cells which had been treated for 10 to 90 min with filter-sterilized culture supernatants (10% v/v) from *V. cholera* or from its isogenic *hlyA* VCC-deficient mutant. BHI broth was used for controls. To evaluate the role of JNK on the VCC-induced activation of c-Jun, cells were pretreated with the JNK inhibitor SP600125 (SP) 30 µM for 2h. Western Blots were performed with specific antibodies against the phosphorylated and total fractions of c-Jun. Protein levels of α-Tubulin were used as loading controls. Below the panels, relative protein levels are shown, with values from BHI-treated cells (10 min) normalized to 1.
